# News and Notes

**Published:** 1910-11

**Authors:** 


### NEWS AND NOTES

Election of officers of the S. A. L. Railway Association of Surgeons, held at Birmingham, Oct 11-12,.

President Surgeon, J. G. Dean, Dawson, Ga.; First Vice-President Surgeon, H. A. Burke, Pettersburg, Va.; Second Vice-President Surgeon, M. L. Wood, Montgomery, Ala.; Third Vice-President Surgeon, T. J. Witherspoon, Charlotte, N. C.; Secretary and Treasurer, J. W. Palmer, Ailey, Ga.; Member Executive Committee, R. L. Harris, Jacksonville, Fla.

Next meeting will be held in Washington, D. C., during Oct., 1911.

---

### CHANGES IN THE PERSONNEL OF THE MEDICAL AT THE UNIVERSITY OF PENNSYLVANIA.

The Trustees of the University of Pennsylvania have announced recently certain changes in the personnel of the teaching staff to go into effect at the beginning of the next academic session, September 1st, 1910.

To fill the Chair of Theory and Practice of Medicine made vacant by the resignation of Dr. James Tyson, Dr. David L. Edsall has been transferred from the Chair of Pharmocology and Therapeutics and the vacancy in the latter will be filled by the appointment of Dr. A. N. Richards, now Professor of Pharmocology in the Medical School of the Northwestern University.

One hundred thousand dollars has been received for the endowment of a Chair of Physiological Chemistry and Dr. Alonzo Englebert Taylor, of the University of Californio, will be its first occupant.

Dr. Richard M. Pearce, of the University and Bellevue Hospital Medical College of New York, has been appointed Professor of Pathology. Dr. Pearce will also direct the work of the Department of Research Medicine recently established by an endowment of $200,000.

Dr. Allen J. Smith, the present dean of the Medical School, will be the occupant of the new Chair of Comparative Pathology and be at the head of the newly instituted courses in Tropical Medicine.

Dr. Paul Lewis, who will have charge of the Laboratory of the Phipps nstitute for the Study, Prevention and Treatment of Tuberculosis, now an integral part of the University, has been elected Assistant Prifessor of Pathology.

---

### THE COST OF WAR.

The New York Peace Society has issued an attractively illustrated circular giving the cost of armed peace in the United States during the past eight years. The total increase during the period amounts to the stupendous sum of $1,072,000,000. The circular pertinently comments on the increase, as follows:

This eight-year increase exceeds the national debt by $158,000, 000.

It exceeds the entire budget of the United States for 1910.

It is twice as much as the highest estimate of carrying out the deep water-ways projects.

It is nearly three times the estimated cost of replanting the 56,000,000 acres of denuded forest land in the United States.

It is three times the estimated cost of the Panama Canal, including purchase price from the French company.

It is three times the cost of carrying out the whole irrigation program contemplated within a generation.

It is probably enough to banish tuberculosis from the United States within a reasonable time, if efficiently used to arouse and assist the people in their fight against this dread disease. More than 160,000 are dying yearly from this cause.

It pays a yearly tax of 1¼ per cent. on the total wages paid in the United States, on the supposition that wages average $600 to the family; and we pay it in the higher price of our goods.

Interest on this sum at 4 per cent. would give an income of $,000 a year fovere to 42,880 failiesa city of 200,000.

The increase for 1908-9 is only $13,000,000 less than all the gifts to charities, libraries, educational institutions and other public causes in 1909, which reached the vast total of $185,000,-000.

Less than twenty years' increase at the present rate only, will absorb the price of the Panama Canal complete systems of deep water-ways of national forests, of irrigation for arid lands, and money to pay the national debt. We propose to issue bonds to carry on such of these projects as we feel able to undertake, while we pour our treasure into army and navy.

Our defenses adequate before 1898, are doubly adequate now. Why not let well enough alone, and buy something besides guns and ironclads? No sane man opposes adequate national defense. We do oppose expenditures required only by personal or national ambition.

---

### PROCEEDINGS OF THE AMERICAN PROCTOLOGICAL SOCIETY.—*Cont.*

---

### LOCATION.

According to Lichtenstein the relative number of instances of these tumors in the different parts of the intestinal tract is indicated in the following arrangement—(the most frequent site of occurrence being in the rectum,)—rectum, ileum, colon, ilia-cecal valve and doudenum.

Malignant degeneration naturally affects the parts named in about the same comparative order of distribution, with the exception of the ileum; this latter being less exposed to insult by reason of the fluid condition of the feces in that region.

It may be noted that these tumors usually manifest themselves in patients between 25 and 35 years old, and the malignant degeneration consequently occurs much earlier on than cancer usually occurs.

About 50% of the cases collected from the literature were under 35 years of age.

A brief summary of the current theories followed.

PATHOLOGICAL FINDINGS.

Several tumors were moved from each case, from the smallest size to the largest. The smaller tumors (that is, those that had recently sprung up) were shown to be composed mostly of granulation tissue, which showed numerous small blood vessels and intersititial fibroblasts. The entire structure is infiltrated by an acute exudate of leucocytes and serum, showing an acute inflammatory process. At the base of the polyp are a few slightly hypertrophied but rather typical glands. The surface epithelium over the polyp shows complete desquamation. The tumor appears to be composed almost entirely of an inflammatory granulation tissue.

DIAGNOSIS—INFLAMMATORY TISSUE, POLYP.

The section through the large polyp, taken from the same individual as the above, but at an advanced stage, showed a growth composed of adenomatous glandular proliferation. There is a narrow peripheral margin in some places about the growth, which shows granulation tissue. The greater part of the growth about the periphery is composed of simply adenomatous glandular proliferation. Throughout the polyp there is an exudate of serum and leucocytes, the latter showing a predominating number of eosinophiles. There is complete desquamation of the superficial epithelium. Some of the glands in the adenoma appear typical; but the greater number are very much larger than those of the rectal mucosa, and are in a condition of marked hyper-secretion.

DIAGNOSIS—ADENOMATOUS POLYP.

These two reports were selected as being typical of what was found in the small and in the well-developed tumor; and go to show an inflammatory starting point with a later proliferation of glandular tissues, which corresponds, to a great extent, with the findings of Lebert and Schwab. Much more might have been learned, had the writer been fortunate enough to have secured a post-mortem on the case that died, as he was confident some of the tumors in the upper part of the sigmoid would have shown carcinomatous degeneration. Again, a section through a growth, down into the bowel might have thrown some further light on the subject.

He hoped to continue the investigation when another opportunity offered.

Reports of cases followed.

---

### "SKIN MANIFESTATIONS OF AMEBIASIS."

By Jno. L. Jelks, M. D., of Memphis, Tenn.

The author had observed cutaneous affections among a number of persons suffering with chronic Amebic infection. In April, 1909, he reported cases before the Annual meeting of the DeSoto County, Mississippi, Medical Society. In May, 1909, be made similar allusions to these conditions before the Annual meeting of the Arkansas State Medical Society. Again in April, of the present year, at the Tennessee State Society in a paper, "Amebiasis, complicated in one instance by Pellagra, in another, by eighteen Adenomata," he referred to these associated conditions.

In one case, observed two years ago, with very Amebic infection and ulceration, the patient had for more than forty years observed the skin lesions, which were erythematous and macular, and at times edematous, depended very greatly upon the condition of the bowel at that time. This patient was returned to her family physician as incurable owing to the scarred, distorted and stenosed condition of the bowel. She has since died, apparently from exhaustion produced by a most extensive desquamative dermatitis.

Another case, which was observed in the winter of 1908-1909, of chronic Amebic ulceration, with liver abscess complicating, presented extensive macular, papular and pustular skin lesions which quickly cleared up under treatment, which was directed solely to the intestinal infection and ulceration.

Recently a case was presented, which had been diagnosed by several able physicians and skin specialists as one of Pellagra. The case presented all symptoms of Amebic infection, which preceded the skin lesious, and the author found the Ent-Ameba Hystolitica in the Muco-purulent material taken from the rectum, and concluded that the condition known as Pellagra may have its solution as to etiology when systematic examinations are made for parastic infections and intestinal conditions.

The author expressed the belief that those may help explain the prevalence of the condition known as Pellagra in the South. A report of six cases was presented in support of his views and he emphasized the singular co-incidental, if not consequential, skin lesions in so many chronic amebic cases which have been observed by him and which responded to treatment directed solely to the intestinal infection and ulceration. He quotes other authority both in this and other countries which are supportive of his views.

---

### "INCONTINENCE FOLLOWING RECTAL OPERATIONS."

By Geo. B. Evans, M. D., of Dayton. Ohio.

We understand the external sphincter to be a flat plane of muscular fibers, elliptical in shape, and intimately adheret to the integument and joining with the pernonei, levator ani and accelator urinae. It is a voluntary muscle and supplied by a branch of the fourth sacral nerve.

The internal sphincter is but a muscular ring, half an inch in breadth, in thickness two lines, and but an aggregation of the involuntary circular fibers of the intestine. Evidently the only true sphincter ani is the external—the internal sphincter ani is not subject to volition—and its sphinteric influence must be largely due to the support afforded it by the practically amalgamated muscles which form the floor of the pelvis and whose main function is the support of the hollow viscera of the pelvic cavity. Would it, therefore, be illigical, to believe that the internal sphincter is not, neither can it be made by any surgical procedure an efficient voluntary constrictor? Certainly, it is true that efficient and satisfactory sphincteric function is dependent on normal support of the bowel by a normal muscular floor, with a normal interdependent power of sphincter muscles, hence any trauma which interferes with muscular function disables proportionately to the extent of the injury.

That incontinence does follow division of the external sphincter, that incontinence does follow division of the internal sphincter, is not denied and when their division becomes a necessity the best way, if there is one, of making the incision should be chosen. Can we hope that ere long there will be a method of cure for fistula-in-ano that will exclude even the possibility of incontinence?

Considering the anatomical conformation of the perineum, the mutual dependence of prefect function, I would admonish those engaged in rectal surgery to not forget that indifferent and multiple injuries (even surgical injuries) should not be indulged in, for fear of a result that would prove more painful and unendurable than the condition which indicated operative interference.

We believe that incontinence can be obviated by relieving the tension of the fibers of the levator ani muscle at their attachment to the externad sphincter, or both the external and the internal sphincter by nicking the fibers of said muscles on eithe rside of the fistulous tract, and thus permitting an incision of the muscle at right angles to the same.

*To be Continued.*

---

### REGULAR MEETING FULTON COUNTY MEDICAL SOCIETY, AUGUST 18, 1910.

REPORTED BY R. R. DALY, M. D.

Dr. Archibald Smith read a paper on the "Care of Puerperal Cases," which appears elsewhere in the Journal.

Dr. Kime said that the only exception he took to the paper was with regard to the use of the douche. For many years he has discarded the use of the douche except where there is infection, or when the hand has been introduced in the uterine cavity. He recommends the use of rubber gloves at delivery to prevent infections of all kinds. Wherever there are even slight tears he sutures at once with plain No. 2 cat-gut stitches. If these are properly made, deep and buried, the mucous membrane falls naturally and successfully into place·

A general point discussed in the Journals and Societies of late is drainage where there is infection. He believes this should be used most carefully and the use of the curette is condemned. The drainage tube is enough for this purpose. He never uses strong solutions in the uterine cavity.

Dr. Roy Blosser said there had been so much improvement in the surgical technique of douches that they should be resumed.

Dr. Duncan said he does not believe in using the douche except in infection. He does use it after instrumental labor, where there are tears in the perinaeum. As to curette, he only uses it after premature labor. Occasionally it is necessary to swab the interuterine wall with a mixture of phenol and iodine.

Dr. Daly said that although this subject was out of his line, he wanted to note that the position of the patient after delivery was assuredly upon the side and face, and the reason for this is that we have not evolved far enough away from our quadrupedal ancestors to lie upon our backs in this condition any more than they would.

Dr. Smith said, in closing, that douches, if used, should not be given by a nurse but by a surgeon as carefully as if he were doing a laporatomy. Tears through mucous membrane only are better left alone. A sponge-holder is better than a curette after labor.

Dr. Wesley Taylor read a paper on "Anterior Poliomyelitis," and presented a case of recovery.

Dr. Toepel said that these cases are often confused with meningitis when the muscles are not all carefully examined. In the treatment those not fully recovered are often taught trades which they can carry on. The treatment consists of keeping the part warm and at rest. It should be tonic at first and then gentle massage, increasing to heavy massage, should be used. Hydrotherapy and electricity should be introduced as soon as possible, and the return to active muscular exercise should not be delayed. The prognosis, as a rule, is good. As to prophylaxis, children should not be allowed to get overheated and suddenly cool by lying on the cold ground.

Dr. Katherine R. Collins said that as to the laboratory side, Flexner and others have proved the infectiousness of the disease.

Monkeys have been given infection and suffered with the typical trouble. Thus, we have yet to learn the pathway which probably is along the lines of cerebro spinal meningitis through the nasal cavity. The virus resembles that of rabies and small-pox rather than the toxin of diphtheria. It is not readily destroyed by cold. Thus far nothing definite has been indicated as to treatment.

Dr. Cartlege asked if in cases of sudden onset there were febrile manifestations. If it were simply hemorrhagic in onset we would not be likely to have constitutional symptoms.

Dr. Taylor replied that he had seen only one case throughout its course. The highest temperature at any time was 100 degrees. There had been no digestive disturbances preceding the onset. In all probability the most of cases have slight temperature rises. As to treatment, one should be careful not to over-treat, although the family wanted something done at once. It is an infectious disease, doubtless, but he has never seen two cases in one family. There is now an epidemic in Pennsylvania, and while he was in Germany, recently, he found an epidemic in one of the provinces there. The prophylaxis would be so indefinite, because of lack of knowledge, except in epidemic, that it would be hard to maintain.

Dr. Kime read a paper on "Indication and Technique in Vaginal Caesarian Section," which appears elsewhere in the Journal.

Dr. McRae said that in all his practice he had never seen a case of Caesarian Section of any kind, or a condition where it was indicated. It simply had been his good fortune not to come in contact with it. Personally, in all operations on the uterus, he prefers the suprapubic route.

Dr. Archibald Smith said that in cancer of the cervix, which was undilatable, the method was excellent. The operation would seem to be useful.

Dr. McRae presented a case of ectopic pregnancy of one month's duration. The tube and appendix were removed with the ovum and membrane intact within the tube. The operation was simple, but he takes pride in having made the diagnosis. He also presented a case of submucous uterine fibroid not recognized by the curette and discovered only after hysterectomy.

